# *QuickStats:* Age-Adjusted Percentage* of Adults Aged ≥18 Years Who Met the 2018 Federal Physical Activity Guidelines for Both Muscle-Strengthening and Aerobic Physical Activity,^†^ by Urbanization Level^§^ — National Health Interview Survey, United States, 2020^¶^

**DOI:** 10.15585/mmwr.mm7127a6

**Published:** 2022-07-08

**Figure:**
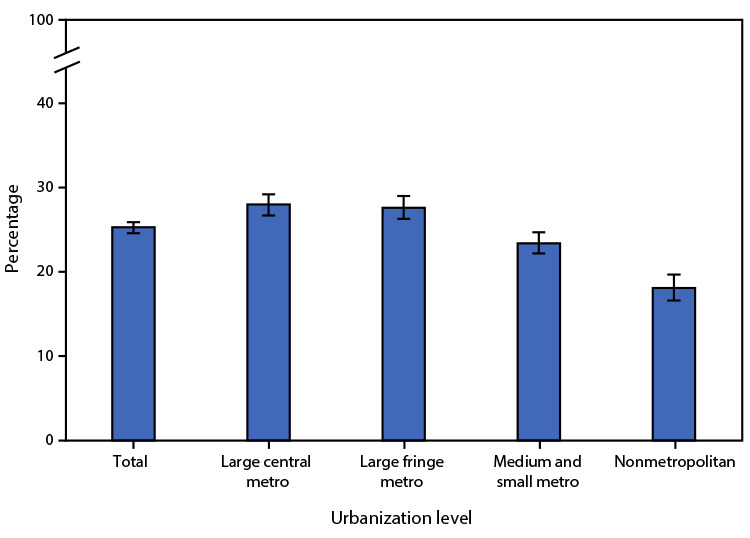
In 2020, 25.3% of adults aged ≥18 years met the 2018 federal physical activity guidelines for both muscle-strengthening and aerobic physical activity. The percentage meeting both guidelines was highest in adults living in large central metropolitan (28.0%) and large fringe metropolitan areas (27.6%), followed by those living in medium and small metropolitan areas (23.4%) and lowest in those living in nonmetropolitan areas (18.1%).

**Source:** National Center for Health Statistics, National Health Interview Survey, 2020. https://www.cdc.gov/nchs/nhis.htm

